# Associations between pathophysiological traits and symptom development in retrospective analysis of V30M and V122I transthyretin amyloidosis

**DOI:** 10.1016/j.ijcha.2025.101663

**Published:** 2025-04-15

**Authors:** Sameer U. Kini, Ha My Thi Vy, Madhav Subramanian, Parasuram M. Krishnamoorthy, Son Q. Duong, Ghislain Rocheleau, Jagat Narula, Ron Do, Girish N. Nadkarni

**Affiliations:** a Scarsdale High School, Scarsdale, NY, United States of America; b The Charles Bronfman Institute for Personalized Medicine, Icahn School of Medicine at Mount Sinai, New York, NY, United States of America; c The Bio Me Phenomics Center, Icahn School of Medicine at Mount Sinai, New York, NY, United States of America; d Department of Genetics and Genomic Sciences, Icahn School of Medicine at Mount Sinai, New York, NY, United States of America; e Washington University School of Medicine, Department of Pathology and Immunology, St. Louis, MO, United States of America; f Department of Medicine, Division of Cardiology, Mount Sinai Hospital, New York, NY, United States of America; g Division of Pediatric Cardiology, Icahn School of Medicine at Mount Sinai, New York, NY, United States of America; h Mount Sinai Heart, Icahn School of Medicine at Mount Sinai, New York, NY, United States of America; i Division of Data Driven and Digital Medicine (D3M), Department of Medicine, Icahn School of Medicine at Mount Sinai, New York, NY, United States of America; j The Hasso Plattner Institute for Digital Health at Mount Sinai, Icahn School of Medicine at Mount Sinai, New York, NY, United States of America

**Keywords:** Heart failure, Symptom onset, Phenome-Wide Association Study (PheWAS), Echocardiogram, Inflammatory markers, African American, Hispanic/Latinx American

## Abstract

**Background:**

The Val30Met (V30M) and Val122Ile (V122I) transthyretin (*TTR*) mutations often beget hereditary amyloid transthyretin amyloidosis (hATTR). Since symptoms are progressively debilitating and potentially fatal if untreated, low survival rates result from late diagnoses of hATTR patients. This retrospective analysis of microarray and biobank data helped establish clinical biomarkers for early hATTR detection.

**Methods:**

In a Portuguese sample of V30M carriers (n = 183), gene profiling identified dysregulated immune markers. Among African Americans (AA) and Hispanic/Latinx Americans (HA) from the Mount Sinai Bio*Me* Biobank (n = 28,718), a case-control style Phenome-Wide Association Study (PheWAS; odds ratio [95% confidence interval]) of V122I for phenotypic and echocardiogram traits (β coefficients [95 % CI]) determined gene pleiotropy.

**Results:**

Among V30M profiles, 96 (52.4%) were symptomatic, expressing upregulated neutrophil activity (p < 10^-16^), IL-6/JAK/STAT3 signaling (p < 10^-3^), and downregulated CD4^+^T cell expression (p = 0.009), compared to their asymptomatic counterparts. In Bio*Me*, 562 (2.0%) were V122I carriers, demonstrating associations with heart failure (1.71 [1.23–2.39]; p = 0.0014), amyloidosis (20.79 [8.42–51.31]; p = 4.67 × 10^−11^), secondary/extrinsic cardiomyopathies (17.73 [7.25–43.37]; p = 2.97 × 10^−10^), peripheral nerve disorders (4.14 [2.42–7.09]; p = 2.26 × 10^−7^), primary angle-closure glaucoma (8.03 [3.15–20.46]; p = 1.27 × 10^−5^), malignant neoplasm of the female breast (4.48 [2.23–9.00]; p = 2.48 × 10^−5^), fracture of tibia and fibula (8.42 [3.25–21.89]; p = 1.19 × 10^−5^), and Carpal tunnel syndrome (2.62 [1.68–4.11]; p = 2.44 × 10^−5^). Echocardiographic presentations included higher LVEDV (15.87 [9.63–22.10]; p = 6.04 × 10^−7^) and LA length (1.52 [0.69–2.35]; p = 3.31 × 10^−4^). Race-stratified associations identified that AA presented more severe cardiac abnormalities than HA.

**Conclusions:**

This study identified inflammatory biomarkers upregulated in symptomatic V30M carriers and phenotypic/echocardiographic traits associated with V122I, representing comorbidities of hATTR pathology. Such markers can provide the basis for future improvements in diagnostic regimes to deliver early therapies.

## Graphical abstract

**Figure:**
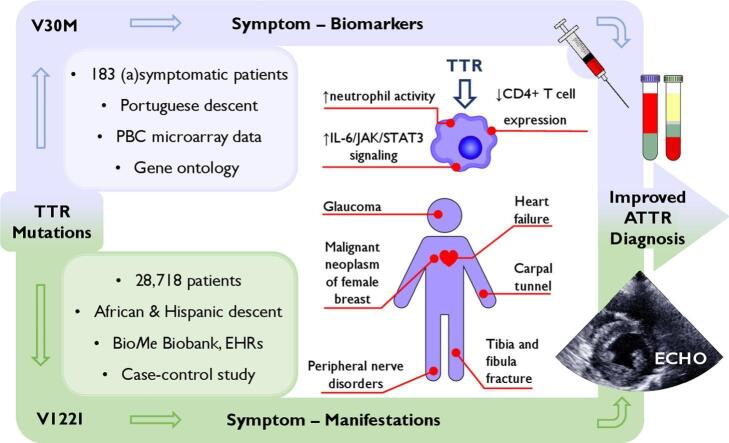
Mutation-stratified workflow identified traits representative of symptom onset in Val30Met (V30M) and Val122Ile (V122I) hereditary amyloid transthyretin amyloidosis (hATTR). For the V30M portion of the study, microarray gene expression profiling data was analyzed to identify the immune signals differentially upregulated in symptomatic V30M compared to their asymptomatic counterparts. In the V122I cohort, biobank data was studied to determine the pleiotropic and echocardiographic traits associated with the V122I mutation. Traits indicative of symptomatology can be measured by clinicians, demystifying hATTR comorbidities and potentially signaling early-stage disease onset.

## 1 Introduction

Hereditary amyloid transthyretin amyloidosis (hATTR) is an understudied and underdiagnosed multisystemic disease [1], [2], [3], for which over 160,000 United States (US) citizens and 1/1000 persons in global endemic regions are genetically susceptible [4], [5]. Over 120 point mutations of the transthyretin (*TTR*) gene can beget hATTR [6], of which the two of the most prevalent are Val30Met (V30M), observed at 0.1% frequency in Portugal [5], and Val122Ile (V122I), observed at 3–4% frequency among African Americans in the US [7]. Both mutations induce misfolds of the liver-produced, homotetrameric TTR protein [8], which is cleaved by proteolytic enzymes into small, toxic oligomers [9], [10], before dissociating into insoluble, monomeric, amyloid fibrils that deposit in tissues [11]. Fibrils accumulate to form larger extracellular structures [12], creating complex disease pathologies through irreparable organ damage [13]. Since symptoms are progressively debilitating and can be fatal if untreated [14], [15], survival rates are poor due to the late diagnoses for most hATTR cases [1], [3], [6].

The V30M mutation is known to cause hATTR through the presentation of polyneuropathy (hATTR-PN) [14]. Toxic V30M TTR oligomers in the bloodstream exhibit a greater affinity than stable TTR for the Receptor for Advanced Glycation End Products (RAGE) on cells involved in NF-κB activation, leading to TNF-α, IL-1β, and IL-6 cytokine release [16], [17]. While elevated toxic oligomer levels generally lead to symptom onset [18], the specific inflammatory changes during initial onset are unclear. Identifying such markers will allow for a better characterization of early TTR instability and enhance disease onset detection before late-stage polyneuropathy.

The V122I mutation has been shown to cause V122I TTR deposition into both the PNS [19] and heart [20], the latter resulting in cardiomyopathy (hATTR-CM). Cardiac presentation of V122I may be responsible for over 10% of congestive heart failure (HF) cases in African Americans (AA) above the age of 65 and is a hypothesized risk factor for Hispanic/Latinx Americans (HA) [21], [22], [23]. Only 11% of V122I carriers are formally diagnosed with hATTR, with a median delay between symptom onset and diagnosis of 3 years, likely due to enigmatic symptom presentations and challenging disease onset detection [22], [24]. Although epigenetic studies have hypothesized that the multitude of methylation sites on the *TTR* gene generate a vast array of disease manifestations [25], [26], V122I phenotypic diversity has remained a mystery due to the lack of large cohort and racially-stratified analyses. Previous phenome association studies have been limited in their ability to capture the surrogate markers of disease onset [27].

Since V30M and V122I are both autosomal dominant, family history is a useful indicator for the potential of disease onset [21], [28]. However, even for many who identified carriers while asymptomatic [29], sudden and insidious symptomatology onset, combined with variable penetrance and pleiotropy, complicates the detection of disease onset [1]. The study seeks to enhance the understanding of hATTR pathology of both mutations by identifying disease markers associated with symptom onset.

Improvement of hATTR prognosis for potential, suspected, or known mutation carriers would involve a combination of a symptom onset detection scheme and a phenome-wide association regimen by identifying the biomedical traits concomitant with disease manifestations. Two datasets were available for this study: the first consisted of transcriptomic peripheral blood samples of Portuguese V30M carriers, while the second contained clinical information of AA and HA V122I carriers. Herein, the objectives to establish biomedical markers for early hATTR detection were two-fold: (1) to identify the gene expression changes in symptomatic versus asymptomatic V30M peripheral blood profiles using gene ontology, differential gene expression, and cell subtype analysis techniques; and (2) to generate a race-stratified resolution of both the general phenotypic traits, as determined by a Phenome-Wide Association Study (PheWAS), and echocardiographic traits associated with V122I carriers compared to non-carriers. The traits identified to be differentially expressed in or associated with hATTR mutations may be concomitant with symptom development and clinically demarcate the early stages of disease onset.

## 2 Methods

With the aim of elucidating the biological characteristics associated with disease onset, this study divided the analyses of the V30M and V122I mutations into four sections: the immune markers upregulated in symptomatic V30M profiles, the phenotypes associated with the V122I mutation, echocardiographic traits associated with the V122I mutation, and two V122I echocardiogram case studies. The data utilized for the V30M portion of this study was public. All authors involved with the V122I portion of this study received full approval from an Institutional Review Board of the Mount Sinai Health System on July 9th, 2022 (STUDY-11-01139), allowing for analysis of patient-level data. Note that all the analyses performed in this study are retrospective in nature, as all data utilized were collected prior to the study's initiation.

### 2.1 V30M profiles

A publicly available database of microarray mRNA transcriptomic profiles, recruited from the peripheral blood cells (PBCs) of asymptomatic and symptomatic Portuguese V30M carriers (n = 183; https://www.ncbi.nlm.nih.gov/pmc/articles/PMC4997237/; Gene Expression Omnibus Accession: GSE67784), was utilized [30]. All PBC microarray data was already pre-processed through a log_2_ transformation of the fold change and quantile normalization of gene expression levels between all selected subjects (Asymptomatic Male, Asymptomatic Female, Symptomatic Male, Symptomatic Female).

#### 2.1.1 Differential expression profiles

The Gene Expression Omnibus tool calculated the genes exhibiting differential expression in symptomatic V30M immune profiles in comparison to the asymptomatic V30M group. Noise in the downstream statistical analyses was minimized by filtering the positively differentially expressed genes down to the top 8,000 highest fold change scores. The biological roles of the filtered genes were examined using EnrichR GO (a gene ontology tool) over the GO_Biological_Processes_2021 library [31], a Gene Set Enrichment Analysis (GSEA; version 4.2.3) [32], and xCell cell subtype analysis [33].

EnrichR program specifications internally accounts for multiple hypothesis testing and adjusted all p-values accordingly. The top 10 most significantly enriched gene pathways were reported in graphical form. Default program specifications for the Gene Set Enrichment Analysis (GSEA) were utilized without modification. The gene pathways with the top 6 highest Normalized Enrichment Score (NES), corresponding to the lowest p-value, were reported. xCell cell subtype analysis was performed in R (version 4.2.0) using default settings, including its deconvolution algorithm which controls for differences in sample compositions. An unpaired *t*-test was conducted between the two groups prior to post-hoc Holm-Bonferroni p-value adjustment, and the results were visualized using GraphPad Prism (version 9.4.1, GraphPad Software, San Diego, California, USA). While the V30M analysis differentiated between asymptomatic and symptomatic V30M, a clinical case-control study regarding the biomedical traits associated with the V122I mutation was performed.

### 2.2 V122I participants

The Icahn School of Medicine at Mount Sinai Bio*Me* Biobank (ISMMSBB) is an EHR-linked database of 56,713 subjects based in New York City and the Tri-State Area between 2007 and 2015. ISMMSBB participants are consenting and are primarily recruited through ambulatory care [22]. Through the ISMMSBB, Regeneron Inc. and Sema4 Inc. sampled, sequenced, and genotyped whole-exome DNA of consenting patients (n = 53,449) [22]. PLINK v2.0 isolated the V122I (rs76992529) mutation [34]. The baseline clinical comorbidities were gathered through EHR data. The overall study analyzed retrospective biobank data in a case-control style, assessing the phenotypes associated with V122I carriers.

#### 2.2.1 Associations with phenotypes including heart failure

For the PheWAS analysis, disease outcomes were extracted from International Classification of Diseases, Ninth Revision, Clinical Modification and International Classification of Diseases, Tenth Revision codes in the ISMMSBB.

For the V122I-HF/echo associations, since V122I hATTR symptoms frequently develop in the seventh decade of life, young V122I carriers have a genetic risk for HF but have not yet required hospitalization [35]. To avoid this bias, HF controls under the age of 60 were removed from the subsequent cardiac analyses.

#### 2.2.2 Echocardiogram associations & case studies

Cardiac morphology was elucidated by the associations between the V122I mutation and echocardiographic traits. SPSS matched V122I carriers with an echocardiogram for age (caliper = 5) and sex with five non-carrier participants (n = 1,121). No age cutoff was used for the continuous outcomes.

Based on the top standardized traits, two deidentified echocardiograms were clinically selected for this study and annotated to contextualize morphological changes in symptomatic V122I hATTR.

#### 2.2.3 Statistical analyses

All analyses using R packages were conducted in RStudio (version 4.2.0) and all analyses using SPSS tools were conducted in IBM SPSS Statistics (Version 28). Assumption verification was performed before any statistical analysis. SPSS calculated significant outliers via Mahalanobis distance (df = 29, α = 0.001), removing 1,210 outlying observations which did not have a significantly different frequency of participants with the V122I mutation (χ^2^ = 0.443; p = 0.506). Ancestral relatedness confounding the V122I mutation was minimized by removing sample kin pairs from the PheWAS input dataset. The ‘PheWAS’ R package performed PheWAS analyses on the entire cohort (n = 25,380) before racial stratification (AA, n = 12,145; HA, n = 13,235) [36].

All association analyses for the PheWAS study were adjusted for age, sex, and the top 20 genetic principal components (PCs) to control for ancestral, racial, and genetic subpopulations [37]. Interaction effects were minimized with the inclusion of cross-multiplication terms between allele, age, sex, and age^2^ as covariates. Each result was confirmed with Bonferroni-corrected, adjusted logistic regression performed separately in the AA and HA racial cohorts. The V122I-HF and posterior regression analyses were additionally adjusted with the clinical comorbidities for HF identified in the baseline population. Inverse-weighted meta-analysis was then performed for all racially-combined cohorts with the ‘meta’ R package [38]. For non-heterogeneous results, the fixed effects models were selected; for heterogeneous results, the random effects models were selected.

SPSS identified the most informative echocardiogram features and controlled for missing data by performing a principal component analysis (PCA) using the default program settings. For each component, the highest correlated echocardiogram trait was picked. Auxiliary traits with the same measurement units as the PCA-selected traits were also chosen to improve and homogenize clinical informativeness of the study. The relative importance of each echocardiogram trait was verified by a 10-fold, 10-repeat rank-of-feature analysis performed by the ‘caret’ R package [39]. Covariate/clinical comorbidity-adjusted linear regression determined the associations between V122I and continuous echocardiogram trait in AA (n = 580) and HA (n = 541) and prior to meta-analysis; conditional logistic regression was used for each binary feature.

The ‘stats’ R package (R Core Team, 2022) performed linear and logistic regression analysis, and the ‘survival’ R package performed conditional logistic regression analysis [40]. Logistic regression results are presented as (odds ratio [95% confidence interval]; p-value) and linear regression results are presented as (coefficient [95% confidence interval]; p-value).

## 3 Results

### 3.1 Baseline characteristics of V30M and V122I study populations

For the V30M portion of the study, the baseline characteristics of the study population were the same as described by Kurian and colleagues [30]. In the V30M hATTR immunology data, the asymptomatic (n = 87) and symptomatic (n = 96) carrier profiles were analyzed. Hierarchical analysis of 33,422 genes identified an upregulation of 16,105 genes in the symptomatic group, of which the top 8,000 were chosen.

For the V122I portion of the study, retrospective data was utilized from the ISMMSBB of consenting participants with corresponding whole-exome sequences (n = 53,449). The two racial cohorts carrying the V122I mutations were selected for study: AA (n = 13,470) and HA (n = 15,248). All V122I carriers (n = 562; AA: n = 436; HA: n = 126) were heterozygous. The differences in baseline comorbidities between the two racial cohorts were not considered clinically significant enough to impact downstream study (Table 1).

**Table 1:** **Baseline characteristics of the AfricanAmerican (AA)and Hispanic/Latinx American (HA)participants in theIcahn School of Medicine at Mount Sinai Bio*Me* Biobank(ISMMSBB) cohort.**

Characteristic	AA (n = 13,470)	HA (n = 15,248)	P-value
Age, mean (SD), years	50.39 (15.59)	50.90 (16.29)	<0.01
Sex, Male, n (%)	5,102 (37.88)	5,715 (37.48)	0.49
Chip, Regeneron, n (%)	8,780 (65.18)	8,546 (56.05)	<0.01
BMI, mean (SD), kg/m^2^	31.15 (8.45)	30.18 (6.80)	<0.01
Diabetes, n (%)	850 (6.31)	994 (6.52)	0.47
Hypertension, n (%)	7,512 (55.77)	7,240 (47.48)	<0.01
Hyperlipidemia, n (%)	4,324 (32.10)	5,380 (35.28)	<0.01
History of Smoking, n (%)	2,738 (20.33)	2,524 (16.55)	<0.01
History of MI/CR, n (%)	619 (4.60)	798 (5.22)	0.012
**Echocardiogram, n (%)**	**4,389 (32.58)**	**4,156 (27.26)**	<0.01
LVEF, mean (SD), %	59.03 (10.99)	59.06 (10.36)	0.81
PWt, mean (SD), cm	1.03 (0.20)	0.98 (0.17)	<0.01
LVEDV, mean (SD), ml	91.13 (35.53)	92.94 (38.16)	<0.01
LVESV, mean (SD), ml	38.31 (27.27)	39.89 (27.76)	<0.01
LVSV, mean (SD), ml	52.84 (16.46)	53.05 (19.77)	0.33
LA Length, mean (SD), cm	5.38 (0.91)	5.36 (1.37)	0.15
E/e’ Ratio, mean (SD)	11.64 (6.44)	11.76 (6.57)	0.12
RV dysfunction, n (%)	668 (15.22)	478 (11.50)	<0.01
RV dilation	539 (12.28)	400 (9.62)	<0.01

AA, African American; HA, Hispanic/Latinx American; Chip, Genome Sequencing Chip; BMI, Body Mass Index; LVEF, left ventricular ejection fraction; PWt, left ventricular end-diastolic posterior wall thickness; LVEDV, left ventricular end-diastolic volume; LVESV, left ventricular end-systolic volume; LVSV, left ventricular stroke volume; LA, left atrium; E/e’, Mitral inflow velocity (E) / mitral annular tissue velocity (e’); RV, right ventricle.

**Fig. 1:**
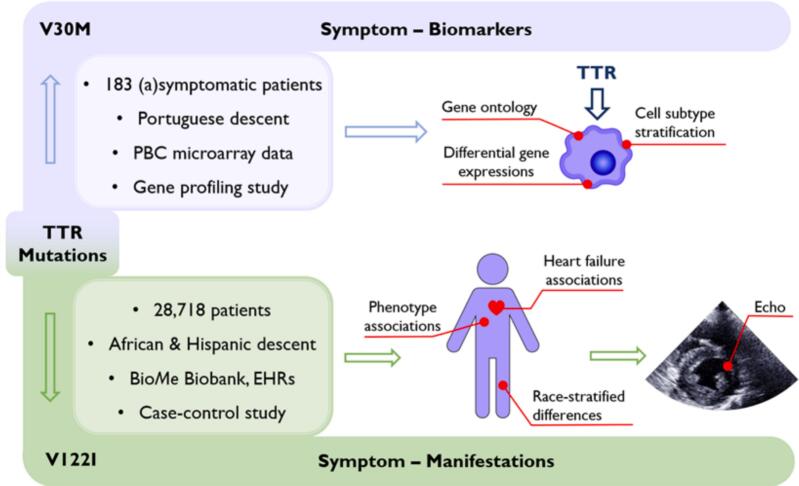
**Mutation-stratified study design to identify traits representative of symptom onset in Val30Met (V30M) and Val122Ile (V122I) hereditary amyloid transthyretin amyloidosis (hATTR).** The V30M baseline characteristics were gathered from Kurian and colleagues while the V122I baseline population was recruited from the Icahn School of Medicine at Mount Sinai Bio*Me* Biobank (ISMMSBB) [30]. In the V30M portion of the study, a gene ontology, differential gene expression analysis, and cell subtype analysis were performed to characterize the immune presentation of symptomatic V30M carriers with respect to their absymptomatic counterparts. In the V122I portion of the study, race-stratified phenotype associations, including heart failure, and echocardiogram trait associations the V122I mutation were assessed to contextualize symptoms with hATTR onset.

The V30M portion of the study identified the gene ontology, differential gene expressions, and cell subtype analysis presented by symptomatic carriers with respect to their asymptomatic counterparts. The V122I portion of the study ascertained a race-stratified analysis of biomedical traits, including heart failure, associated with carriers as well as specific echocardiographic features of V122I symptom presentation in the hospital setting (Fig. 1).

### 3.2 Gene profiling of symptomatic V30M

V30M hATTR immune profiles were analyzed using EnrichR GO to characterize the functions of differentially expressed genes in symptomatic versus asymptomatic profiles. Symptomatic V30M carriers exhibited a greater expression of genes involved in neutrophil activity (p < 10^−16^), extracellular matrix organization (p < 10^−6^), and type I interferon signaling (p < 10^−4^) compared to asymptomatic V30M carriers (Supplemental Fig. 1).

**Fig. 2:**
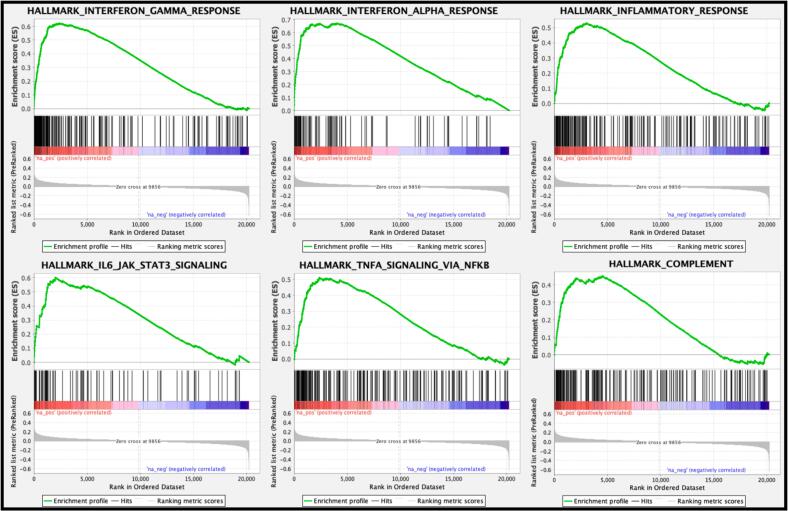
**Gene set enrichment analysis (GSEA) of the upregulated genes in symptomatic V30M exhibited differential immune activity.** The fold change of each gene differentially upregulated in symptomatic versus asymptomatic Val30Met (V30M) was used to calculate the most enriched biological pathways. The top 6 pathways were reported based on highest normalized enrichment score (NES). All p-values were internally corrected for multiple hypothesis testing in the GSEA software (version 4.2.3).

Differential immunological processes were further elucidated by a GSEA, which detected the top six upregulated gene sets: interferon-gamma (IFN-γ) response (Normalized Enrichment Score (NES) = 2.80; p < 0.001), IFN-α response (NES = 2.75; p < 0.001), inflammatory response (NES = 2.43; p < 0.001), IL-6/JAK/STAT3 signaling (NES = 2.40; p < 0.001), TNF-α/NF-κB signaling (NES = 2.29; p < 0.001), and complement system (NES = 2.02; p < 0.001; Fig. 2).

**Fig. 3:**
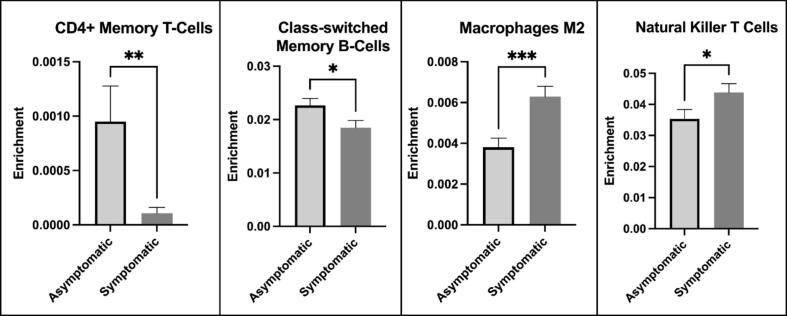
**Xcell analysis localized the differently expressed symptomatic compared to asymptomatic genes to four leukocytes.** xCell enrichment scores of cell mRNA expression were compared using two-sided, unpaired t-tests reporting the mean (SEM) followed by post hoc Holm-Bonferroni p-value corrections (*p < 0.05, **p < 0.01, ***p < 0.001).

The cell-subtype specificity of immune markers hypothesized to induce leukocyte morphological changes were revealed by the xCell analysis, as the upregulated genes were localized to M2 macrophages and natural killer T cells. There was a net downregulation of genes primarily expressed in CD4^+^ memory T cells and class-switched memory B cells (Fig. 3).

Beyond the discovery of peripheral blood markers accompanying symptom onset, the second phase of this study sought to reveal the biomedical traits associated with hATTR in known carriers. The pleiotropy of the V122I mutation was elucidated through a retrospective case-control study conducted over the clinical ISMMSBB dataset.

### 3.3 Phenome-wide association study of V122I

**Fig. 4:**
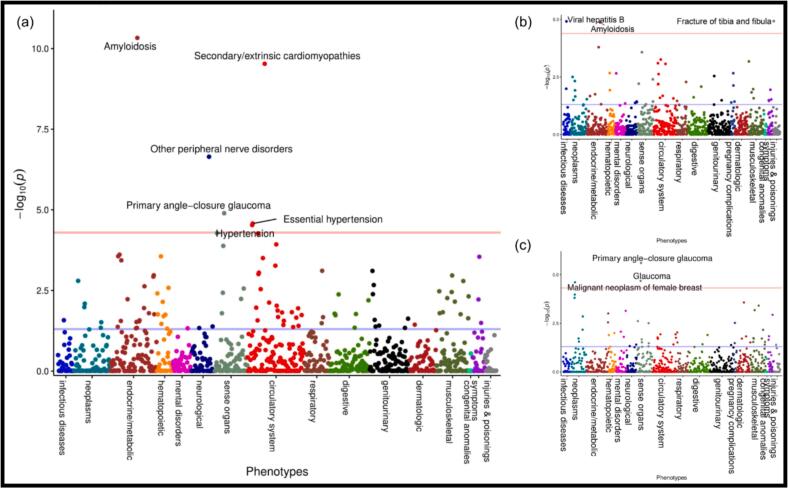
**Phenome-wide associationstudy (PheWAS)of the Val122Ile (V122I) mutation.** The red horizontal lines represent the Bonferroni-corrected p-values. All x-axes are identical. (a) Associated phenotypes over the combined V122I cohort were identified (p > 4.98 × 10^-5^). Amyloidosis and secondary/extrinsic cardiomyopathies were separately tested for association with V122I. (b) African American (AA)-associated phenotypes were identified (p > 3.97 × 10^-5^). (c) Hispanic/Latinx American (HA)-associated phenotypes were identified (p > 4.74 × 10^-5^). Incorrectly fitted models (hypertension, essential hypertension, viral hepatitis B, and fracture of tibia and fibula (for HA only)) were removed from posterior analyses and necessitated the grouping of primary angle-closure glaucoma and glaucoma as a single outcome. Since carpal tunnel syndrome was previously described in a lower sample study of the same biobank to be present in V122I carriers [19], [22], it was added as an outcome of interest.

In the ISMMSBB cohort, a PheWAS of 1,856 hierarchically arranged diagnostic phenotypes, adjusted for genetic ancestry using principal components, was performed over all individuals. The associations between the V122I mutation and diagnostic phenotypes included amyloidosis (20.79 [8.42–51.31]; p = 4.67 × 10^−11^), secondary/extrinsic cardiomyopathies (17.73 [7.25–43.37]; p = 2.97 × 10^−10^), other peripheral nerve disorders (4.14 [2.42–7.09]; p = 2.26 × 10^−7^), primary angle-closure glaucoma (8.03 [3.15–20.46]; p = 1.27 × 10^−5^), malignant neoplasm of female breast (4.48 [2.23–9.00]; p = 2.48 × 10^−5^) and fracture of tibia and fibula (8.42 [3.25–21.89]; p = 1.19 × 10^−5^; Fig. 4).

The impact of racial ancestry was elucidated through adjusted logistic regressions performed in the AA and HA cohorts: 2 PheWAS-determined traits were significantly associated with V122I (at nominal p-value < 0.05) only in AA, 2 were significantly associated only in HA, and 3 (including Carpal tunnel syndrome added during posterior analyses) were associated with both cohorts (Table 2). Since the strongest associations were cardiac diseases pathognomonic to amyloidosis, the association between V122I and heart failure (HF) was specifically investigated.

**Table 2:** **ISMMSBB cohort race-stratified associations between V122I and diagnostic phenotypes.** Adjusted logistic regression was performed over the African Americans (AA) and Hispanic/Latinx Americans (HA) racial cohorts.

	**African Americans**		**Hispanic/Latinx Americans**	
**Phenotype**	**OR [95 % CI]**	**Adj. p-value**	**OR [95 % CI]**	**Adj. p-value**
Amyloidosis	25.38 [8.45–74.93]	3.75 × 10^−9^	52.48 [8.01–282.57]	7.30 × 10^−6^
Secondary cardiomyopathies	7.61 [2.50–19.71]	9.01 × 10^−5^	26.17 [3.18–128.17]	2.42 × 10^−4^
Other peripheral nerve disorders	2.30 [1.44–3.58]	3.29 × 10^−4^	2.73 [1.10–6.19]	0.0214
Primary angle-closure glaucoma	2.03 [0.60–5.22]	0.189	6.63 [2.41–15.40]	4.97 × 10^−5^
Malignant neoplasm of the female breast	1.29 [0.61–2.51]	0.473	5.41 [2.06–13.08]	3.05 × 10^−4^
Fracture of tibia and fibula^a^	3.11 [1.28–6.40]	4.97 × 10^−3^	N/A	N/A
Carpal tunnel syndrome	2.29 [1.33–3.79]	1.90 × 10^−3^	3.85 [1.53–8.87]	2.45 × 10^−3^

OR, Odds Ratio; CI, confidence interval; Adj., adjusted after controlling for the covariates.

a Incorrectly fitted model (fracture of tibia and fibula in HA) due to separation.

### 3.4 Association between V122I and heart failure

In the ISMMSBB cohort, V122I carriers constituted 65 of 2,549 (2.55%) HF cases and 497 of 26,169 (1.90%) non-HF controls (χ^2^ = 5.1278; p = 0.024). Non-HF controls under the age of 60 were excluded (n = 10,053). The adjusted fixed effects meta-analysis model established that V122I was associated with HF (1.71 [1.23–2.39]; p = 0.0014). Adjusted logistic regression found that the association between V122I and HF was maintained in AA (1.67 [1.13–2.50]; p = 0.009) but failed to be detected in HA (1.83 [0.94–3.35]; p = 0.064; Table 3).

**Table 3:** **ISMMSBB cohort race-stratified associations between V122I and HF.** After adjusting for covariates and clinical comorbidities, logistic regression was performed separately in African Americans (AA) and Hispanic/Latinx Americans (HA) before inverse variance weighted meta-analysis generated a combined cohort statistic.

Ancestry	Controls	Cases	OR	95% CI	Adj. p-value
African	3531	1271	1.67	1.13–2.50	0.009
Hispanic/Latinx	3973	1278	1.83	0.94–3.35	0.064
Meta-analysis	7504	2549	1.71	1.23–2.39	0.0014

OR, odds ratio; CI, confidence interval; Adj., adjusted after controlling for all covariates and clinical comorbidities.

Due to the enigmatic nature of the high-level connection between hATTR and congestive HF, further study was conducted to elucidate the particular relationship between V122I and specific cardiac manifestations.

### 3.5 Association between V122I and echocardiographic traits

PCA and rank-of-feature analysis resolved the most informative echocardiogram features: left ventricular ejection fraction (LVEF; %), LV end-diastolic posterior wall thickness (PWt; cm), LV end-diastolic volume (LVEDV; biplane view; ml), LV end-systolic volume (LVESV; biplane view; ml), LV stroke volume (LVSV; biplane view; ml), left atrial (LA) length (2 chamber view; cm), mitral inflow velocity (E)/mitral annular tissue velocity (e′) ratio, right ventricular (RV) dysfunction, and RV dilation.

In the matched cohort of ISMMSBB participants with reported echocardiograms, V122I was associated with some of the selected echocardiogram traits. The cardiac abnormalities were localized to the LV and LA, as V122I carriers displayed an increase in chamber volumes at all major rhythmic points and a large LA size. There was no significant decrease in LVEF (Table 4).

**Table 4:** **Matched ISMMSBB cohort associations between V122I and echocardiogram traits.** The combined cohort statistics were generated using the fixed effects model^a^ in inverse-variance weighted meta-analysis.

Echo trait (n = 1,121)	β coef. [95% CI]	Adj. p-value^a^
LVEF^a^	−1.59 [−5.74–2.55]	0.451
PWt	0.0970 [0.0341–0.160]	2.53 × 10^−3^
LVEDV	15.87 [9.63–22.10]	6.04 × 10^−7^
LVESV	5.35 [2.27–8.43]	6.50 × 10^−4^
LVSV	10.54 [7.05–14.02]	3.08 × 10^−9^
LA length	1.52 [0.69–2.35]	3.31 × 10^−4^
E/e’ Ratio	−0.0969 [−1.51–1.32]	0.893
RV dysfunction^b^, ^c^	1.33 [0.26–6.86]	0.734
RV dilation^c^	1.62 [0.87–2.99]	0.127

β coef., β coefficient; CI, confidence interval; Adj., adjusted after controlling for the covariates and clinical comorbidities; LVEF, left ventricular ejection fraction; PWt, left ventricular end-diastolic posterior wall thickness; LVEDV, left ventricular end-diastolic volume; LVESV, left ventricular end-systolic volume; LVSV, left ventricular stroke volume; LA, left atrium; E/e’, Mitral inflow velocity (E) / mitral annular tissue velocity (e’); RV, right ventricle.

a The significantly reported p-values survived Bonferroni correction for cardiac traits (p < 0.005).

b Random effects model was used as heterogeneity (LVEF: Q = 4.33, I^2^ = 76.9 % [0.0−94.7%]; p = 0.0374; RV dysfunction: Q = 5.46, I^2^ = 81.7% [22.5−95.7%]; p = 0.0194) was detected in the association with V122I among the African American (AA) and Hispanic/Latinx American (HA) racial cohorts.

c For binary outcome variables, the odds ratio (in place of the coefficient) and 95% confidence interval were reported.

The interplay of racial ancestry was investigated through appropriate adjusted regression analysis between V122I and the echocardiogram features, which revealed a significant decrease in LVEF. LV morphological changes were elucidated with conditional multinomial logistic regression, which determined the association between V122I and concentric LV hypertrophy in AA (1.88 [1.10–3.23]; p = 0.021) and HA (0.66 [0.15–2.84]; p = 0.573). V122I was associated with an increased odds of being diagnosed with RV dysfunction and dilation in AA but not HA. The reported differences were confirmed by the ratio of odds ratios (ROR = 5.39 [2.52–42.54]; p = 1.94 × 10^−2^). The effect of RV dysfunction on pathophysiological mechanics was elucidated with conditional logistic regression, which discovered an association between V122I and generalized/nonspecific pulmonary heart disease in AA (1.81 [1.07–3.05]; p = 0.027) but not in HA (0.87 [0.24–3.10]; p = 0.826). Similarly, though the association between V122I and LVESV was higher in the HA cohort, V122I was only associated with LVEF in AA. All other traits involving volumetric LV and LA measurements, except E/e’ ratio, were significantly associated with V122I in both racial cohorts (Table 5).

**Table 5:** **Matched ISMMSBB cohort race-stratified associations between V122I and echocardiogram traits.** Appropriate regression was performed for African Americans (AA) and Hispanic/Latinx Americans (HA).

	African Americans (n = 580)		Hispanic/Latinx Americans (n = 541)	
Echo Trait	β coef. [95% CI]	Adj. p-value	β coef. [95% CI]	Adj. p-value
LVEF	−4.27 [-8.45 − −0.10]	0.0459	−2.69 [-6.85–1.48]	0.207
PWt	0.13 [0.02–0.24]	0.0177	0.080 [0.001–0.158]	0.0495
LVEDV	14.0 [6.31–21.8]	3.77 × 10^-4^	19.9 [8.59–31.2]	6.38 × 10^-4^
LVESV	4.60 [0.80–8.41]	0.0177	6.97 [1.39–12.5]	0.0149
LVSV	9.43 [5.12–13.7]	1.91 × 10^-5^	12.9 [6.61–19.2]	7.42 × 10^-5^
LA length	1.76 [0.49–3.02]	0.00679	1.33 [0.19–2.47]	0.0223
E/e’ Ratio	1.12 [-1.86–4.11]	0.460	−0.47 [-2.12–1.18]	0.576
RV dysfunction^a^	2.81 [1.50–5.29]	0.0013	0.52 [0.15–1.85]	0.313
RV dilation^a^	2.04 [1.04–4.01]	0.0376	0.47 [0.10–2.21]	0.340

β coef., β coefficient; CI, confidence interval; Adj., adjusted after controlling for the covariates and clinical comorbidities.

LVEF, left ventricular ejection fraction; PWt, left ventricular end-diastolic posterior wall thickness; LVEDV, left ventricular end-diastolic volume; LVESV, left ventricular end-systolic volume; LVSV, left ventricular stroke volume; LA, left atrium; E/e’, Mitral inflow velocity (E) / mitral annular tissue velocity (e’); RV, right ventricle.

a For binary outcome variables, conditional logistic regression calculated the association. The odds ratio (in place of the coefficient) and 95% confidence interval were reported.

Since V122I carriers presented enigmatic LV pathologies, high clinical expressions of the intricate cardiac traits were illustrated by two case studies.

### 3.6 Echocardiogram case studies of V122I

The visual mechanics of V122I hATTR cardiac complications were highlighted by two echocardiogram studies: a 74-year-old African-American male and a 66-year-old Hispanic/Latinx American female. For the AA man, dilated LA area (36 cm^2^; reference (ref): 11.4–25.0 cm^2^) and hypertrophic LV mass/body surface area (115.74 g/m^2^; ref: 39.8–109.8 g/m^2^) contributed to an excessive LVESV (109 ml; ref: 15.3–61.7 ml) and diminished LVEF (27%; ref: 53.5–73.1%), indicating severe diastolic and systolic dysfunction [41]. Abnormal RA area (38.0 cm^2^; ref: 10.3–21.9 cm^2^) presented with impaired RV function (Supplemental Fig. 2A, B).

For the HA woman, a hypertrophic LV (100.6 g/m^2^; ref: 33.3–98.9 g/m^2^) obstructed LV contraction and resulted in a low LVEF (50%; ref: 53.2–76.4%). The LA area (28 cm^2^; ref: 9.6–22.0 cm^2^) and RA area (23.0 cm^2^; ref: 7.4–19.0 cm^2^) were abnormally high. The RV size was noted by the physician to be normal, corresponding with the association between V122I and RV dysfunction and dilation in AA but not HA (Supplemental Fig. 2C and D).

Echocardiogram data demonstrated the cardiac morphological changes with exemplar symptomatic V122I carriers. Hallmark cardiological shifts, such as biatrial enlargement, concentric LV hypertrophy, and right ventricular dysfunction (in AA only), were clinical markers pathognomonic of amyloidogenic processes.

## 4 Discussion

The enigmatic pathogenesis of V30M and V122I hATTR leaves early disease diagnosis as a prominent challenge for clinicians. With the aim of improving symptom onset detection for suspected or known V30M or V122I carriers, the biological mechanisms and pleiotropic effects of these mutations were measured in the form of immune signatures due to TTR-RAGE binding in leukocytes of symptomatic V30M and concomitant symptom presentations in V122I carriers.

While previous small and *in vitro* experiments have disagreed over the role of inflammation during the first stages of mutant TTR production [16], [17], [42], [43], [44], this study’s findings indicate that IFN-α and IFN- γ response, TNF-α activation through NF-κB, as well as IL-6/JAK/STAT3 signaling are implicated in early V30M pathogenesis. TTR likely damages endothelial cells which release chemoattractants for neutrophils and monocytes that differentiate into macrophages [45], [46], explaining the concomitant detection of IFN-γ response and M2 macrophage polarization. Regardless of whether amyloidogenic stress triggers greater levels of inflammatory gene expression, leukocyte population increase, or perhaps both, the cell-cytokine relationships demonstrate a striking trend of enhanced innate immune activity and diminished active immune response.

Since early-stage, *in vitro* TTR expresses the greatest differential IL-6 activation in monocytes compared to CD4^+^ T cells [15], [43], it is possible that high IL-6 but low CD4^+^ T cell activation in the peripheral blood foreshadows the primary stages of TTR misfolding. The immune markers detected in this study, some of which have been previously found in sural nerve and salivary gland biopsies after amyloid damage to tissues [16], reside in the peripheral blood and thus may be indicators of early disease onset for those suspected or known to carry V30M. The V30M analysis for leukocytes may extend to the V122I mutation as well since V122I *TTR* fibrils may have also have a tendency to bind RAGE on myocardium-resident macrophages [20], [47], [48], [49]. Properly cross-applying the V30M results to V122I requires further investigation to confirm the causative mechanisms of mutant TTR deposition into the various bodily organs.

The early stages of hATTR are also characterized by pathophysiological symptom presentations. The seven hallmark phenotypes identified in the race-stratified PheWAS analysis could both be concomitant with symptomatic development and, among those suspected or known to be V122I carriers, demarcate the early stages and potential comorbidities in hATTR pathology [21]. While previous studies have reported oculoleptomeningeal amyloidosis in certain demographics such as Caucasian women or V30M carriers [50], [51], [52], the results herein indicate retinal manifestations of the V122I mutation in AA and HA. Similarly, while *in vitro* and murine models have hypothesized that V122I causes osteoarthritis and subsequent inflammation [53], [54], [55], [56], the association found between V122I with fractures of the tibia and fibula provides greater evidence of a translation into negative clinical outcomes. Furthermore, since hATTR clinical trials have reported heterogeneous levels of cancer prevalence [57], [58], [59], the association between V122I and malignant breast tumors in women suggests that cancer is a frequent comorbidity for symptomatic HA V122I carriers. Elucidating the phenotypes concomitant with disease onset enhances the medical understanding of hATTR natural history and can assist with early detection when monitoring suspected or known V122I carriers.

Cardiac V122I symptoms are also inextricably related to structural abnormalities of the heart. The PheWAS and echocardiogram findings establish a race-inclusive tabulation of hATTR phenotypes which validate previous single-ancestry studies while also advancing scientific understanding of the different cardiac morphologies between V122I carriers of varying demographic characteristics. Most previous echocardiographic studies of V122I are low sample size, anecdotal, or non-diverse [60], [61], [62], [63]. The largest ongoing, single-cohort, echocardiographic study of the various hATTR mutations across Europe, known as the Transthyretin Amyloidosis Outcomes Survey (THAOS), reported a general association between hATTR and mildly depressed LVEF as well as biventricular hypertrophy [64]. This study corroborates similar results in a large AA American and HA cohort with a much higher frequency of the V122I mutation. The previous ISMMSBB study only reported a marginally significant association between V122I and LVEF and LV posterior wall thickness in individuals under 45 years of age, a heuristically chosen ceiling [22]. The current findings demonstrate age- and sex-matched associations between V122I and more echocardiogram traits, including RV dysfunction and dilation in AA, which provide a qualitatively more holistic and descriptive cardiomyopathic program.

HF associated with V122I indicates that carriers presented an abnormal LV leading to excess LVEDV and LVESV as well as enlarged LA size [65], [66], [67], [68], [69]. Long periods of amyloid fibril deposition into the myocardium may have caused the observed concentric hypertrophy and thickened posterior walls, leading to a decreased contractile force and LVEF, as well as an increased in LVESV [70], [71]. An already overfilled LV receiving more blood from the LA during the diastolic cycle likely explains the observed increase in LVEDV [72]. Amyloidogenic stress can result in increased LV diastolic filling pressures as well as LA remodeling and enlargement [73], [74], [75]. The vicious cycle of impaired muscular relaxation and reduced contractile force results in both diastolic and systolic HF. The implication of V122I in concentric LV hypertrophy corroborates the findings of HF with reduced EF in AA, indicating that pathogenesis may be distinct based on genetic ancestry. Similarly, the fact that V122I had a higher coefficient of association with LVESV, yet significantly reduced LVEF was only observed in AA, indicates either a greater power in the AA analysis due to allele prevalence or that HA V122I carriers did not experience a lower LV blood ejection during systole. The difference between the meta-analyses and race-stratified results demonstrates the complex interplay between genetic background and the propensity for hATTR disease type, as well as the need for earlier disease treatments.

Various hATTR-PN therapies have been approved by the U.S. Food and Drug Administration (FDA), such as Tafamidis and Diflunisal, which inhibit TTR protein dissociation, or Patisiran and Inotersen, which target the mRNA coding for the TTR protein [65]. The only current FDA-approved treatment for hATTR-CM is Tafamidis [24]. Recent clinical trials have demonstrated the efficacy Sodium-Glucose Cotransporter 2 Inhibitors (SGLT2i) in slowing the progression of cardiomyopathy regardless of EF in hATTR, which has traditionally been excluded from SGLT2i clinical trials [76]. The role of genetic ancestry in HF with preserved EF presentations of hATTR-CM found in this study illuminate the need for future research into the complex interactions between SGLT2i treatment and cardiomyopathy outcomes. Since the mean time between symptom onset and diagnosis of V30M early-onset hATTR is 2.8 years [77], and as few as and 11% of V122I carriers are diagnosed for whom the median survival post-diagnosis is 2.6 years [22], [78], [79], improving the early detection of disease onset via a holistic schema of concomitant immunological, phenotypic, and echocardiographic markers is paramount to maximizing prognosis. Demystifying the true mechanics of hATTR onset requires further longitudinal analysis.

## 5 Limitations

Since the data analyzed in this study are retrospective in nature, conclusions regarding the longitudinal history of hATTR pathogenesis are difficult to draw as it is challenging to separate changes in disease progression due to the V30M or V122I mutations from ulterior causes. For the V30M portion of the study, it is not clear whether symptom onset causes inflammation or vice versa, and it is likely that both are at play. For the V122I portion of the study, participants recruited in hospital settings likely exhibit a greater prevalence of background comorbidities than the general population. This may have biased the disease controls in the non-V122I cohort compared to their V122I counterparts, many of whom would be eventually hospitalized anyway, leading to lower effect sizes measured in this study than in the general population. Additionally, the described associations would be most clinically applicable when the patient is suspected or known carry the V30M or V122I mutations.

## 6 Conclusions

The V30M and V122I mutations are largely dormant until the rapid onset of progressively debilitated systemic insult. Since early therapy is paramount to optimizing prognosis, the discoveries herein describe influential immune markers present in symptomatic V30M carriers and hallmark biomedical traits associated with V122I in AA and HA. Innate immune system overexpression and concomitant lymphocyte downregulation was found to be marker of hATTR pathology in symptomatic V30M carriers. These specific leukocytes may be the primary location or drivers of inflammation in hATTR and could demarcate the risk or onset of disease. This study also identified seven hallmark phenotypes associated with both AA and HA V122I carriers, with race-specific differences appearing in lesser-known comorbidities of hATTR pathology as well as in the echocardiographic presentations of cardiomyopathy. More severe changes in cardiac symptoms were found in AA V122I carriers compared to controls than HA, indicating the complex role of genetic background in hATTR disease mechanics.

Biological processes that develop concomitantly with symptom onset both reveal the early stages and elucidate potential comorbidities of hATTR, which helps enhance disease onset detection for those already suspected or known to harbor the mutation. Indications of early onset can be gathered from bloodwork, EHR review, and echocardiographic data. Since a cause-effect relationship cannot be conclusively drawn between V122I and the discovered phenotypes in a case-control setting, the predictive applications of the results hererin should be further investigated in a randomized, longitudinal study that identifies carriers at the prime age for genetic penetrance. Following up on physiological outcomes would best establish the predictive probabilities and survival analyses of the associations discovered in this study. Given the understudied and enigmatic nature of hATTR, these immunological, phenotypic, echocardiographic findings provide the basis for future improvements in disease onset detection, risk-stratification, and the initiation of early therapies to prevent end-stage disease progression for underdiagnosed patients in the United States and across the globe.

## CRediT authorship contribution statement

**Sameer U. Kini:** Conceptualization, Methodology, Software, Validation, Formal analysis, Investigation, Writing – original Draft, Writing – eview & editing, Visualization. **Ha My Thi Vy:** Resources, Methodology, Formal analysis, Data curation. **Madhav** **Subramanian:** Validation, Resources, Methodology, Formal analysis, Visualization. **Parasuram M. Krishnamoorthy:** Visualization, Supervision, Methodology, Conceptualization. **Son Q. Duong:** Data curation, Resources, Visualization. **Ghislain Rocheleau:** Validation, Software, Methodology, Investigation, Data curation. **Jagat Narula:** Writing – review & editing, Validation, Supervision, Investigation, Conceptualization. **Ron Do:** Writing – review & editing, Supervision, Investigation, Formal analysis, Conceptualization, Funding acquisition, Project administration. **Girish N. Nadkarni:** Writing – review & editing, Supervision, Resources, Conceptualization, Funding acquisition, Project administration.

## Funding

Dr. Nadkarni and Dr. Do are supported by 10.13039/100000002National Institutes of Health (NIH) grant R01-HL155915.

## Declaration of competing interest

The authors declare the following financial interests/personal relationships which may be considered as potential competing interests: Dr. Nadkarni and Dr. Do are supported by National Institutes of Health (NIH) grant R01-HL155915. Dr. Nadkarni reports consultancy agreements with AstraZeneca, BioVie, GLG Consulting, Pensieve Health, Reata, Renalytix, Siemens Healthineers and Variant Bio; research funding from Goldfinch Bio and Renalytix; honoraria from AstraZeneca, BioVie, Lexicon, Daiichi Sankyo, Menarini Health and Reata; patents or royalties with Renalytix; owns equity and stock options in Pensieve Health and Renalytix as a scientific cofounder; owns equity in Verici Dx; has received financial compensation as a scientific board member and advisor to Renalytix; serves on the advisory board of Neurona Health; and serves in an advisory or leadership role for Pensieve Health and Renalytix.

Dr. Do reports consultancy agreements with Variant Bio; research funding from Goldfinch Bio; is a consultant and equity holder (pending) in Pensieve Health as scientific cofounder. 

All other authors and co-authors have no disclosures to report.
