# Supplementary material for: Associations between pathophysiological traits and symptom development in retrospective analysis of V30M and V122I transthyretin amyloidosis
Source: Int J Cardiol Heart Vasc. 2025 Apr 15;58:101663. doi: 10.1016/j.ijcha.2025.101663 (PMC12019459; doi:10.1016/j.ijcha.2025.101663)

**Supplemental Figure 1**. EnrichR GO analyses of differentially expressed genes in V30M hATTR symptomatic (n=96) versus asymptomatic (n=87) profiles


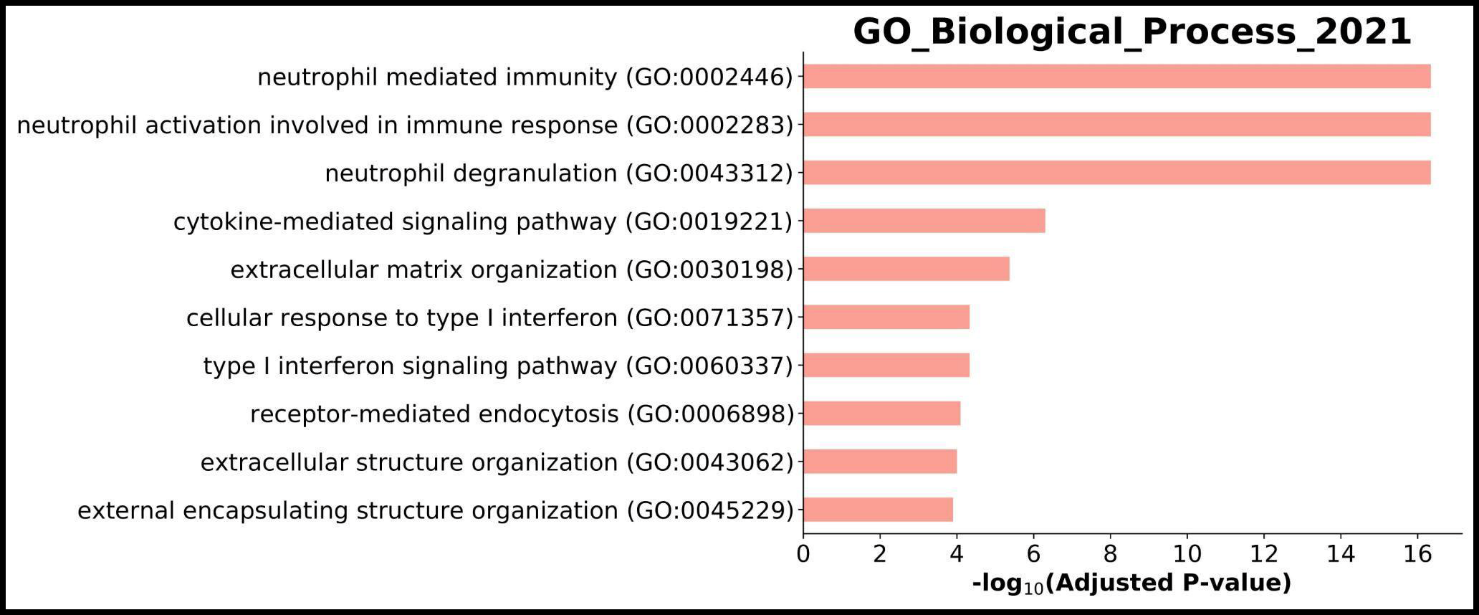


**Supplemental Figure 2**. Echocardiograms of symptomatic V122I carriers. (A, B) A symptomatic 74-year-old African American male V122I carrier. Parasternal long-axis view illustrating: (I) thick LV posterior wall; (II) LA enlargement; (III) dilated RV. (B) End-systolic snapshot of apical 4-chamber cine-loop displaying: (I) thickened left ventricular (LV) walls; (II) dilated left atrial (LA) cavity size; (III) dilated right atrium; (IV) abnormal RV dilation. (C, D) Echocardiograms of a symptomatic 66-year-old Hispanic American female V122I carrier. (C) Apical 4 chamber view portraying: (I) normal left ventricular (LV) cavity size; (II) enlarged left atrial (LA) area; (III) enlarged right atrial (RA) area. (D) Parasternal short-axis view exemplifying: (I) thick LV walls due to concentric hypertrophy; (II) normal RV cavity size. The white dots along the left edges demarcate centimeters.


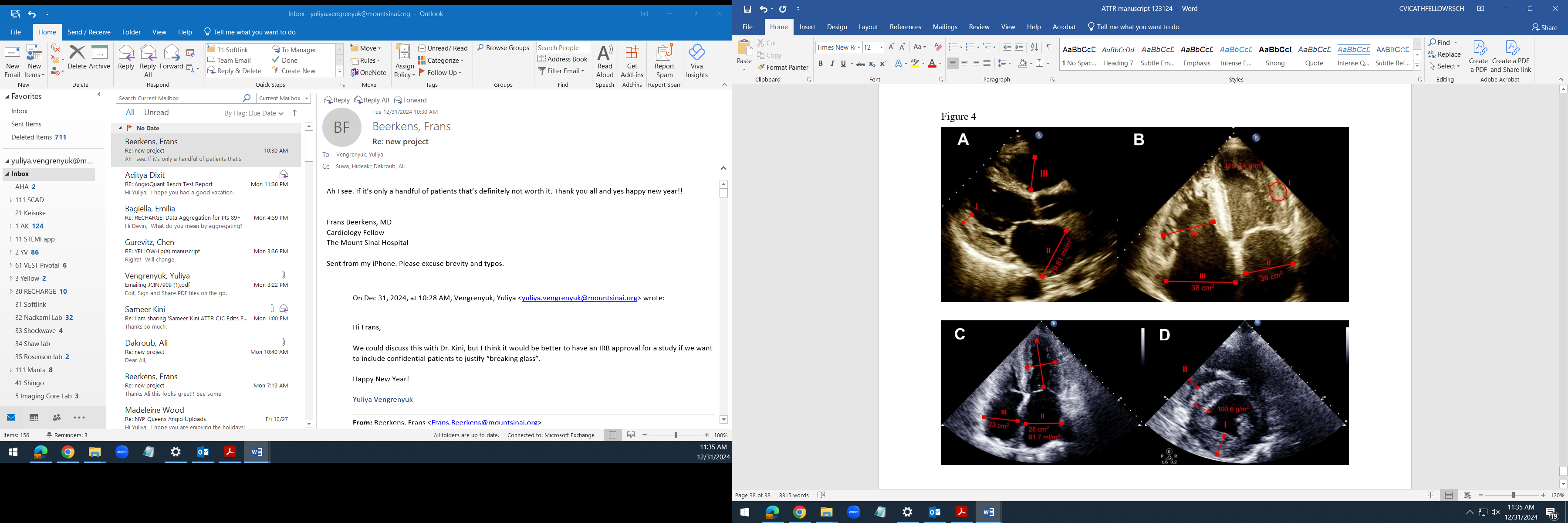

Supplement: Supplementary Data 1 [file mmc1.docx]
